# Influence of different types of pulp treatment during isolation in the obtention of human dental pulp stem cells

**DOI:** 10.4317/medoral.20957

**Published:** 2016-03-06

**Authors:** Jose Viña-Almunia, Consuelo Borras, Juan Gambini, Marya El Alamy, Miguel Peñarrocha, Jose Viña

**Affiliations:** 1 Doctor in dentistry. Collaborating professor of the Master in Oral surgery and Implant Dentistry, Stomatology Department, Faculty of Medicine and Dentistry, University of Valencia, Spain; 2 Full professor of Physiology, Physiology Department, Faculty of Medicine and Dentistry, University of Valencia, Spain; 3 Associated professor, Physiology Department, Faculty of Medicine and Dentistry, University of Valencia, Spain; 4 Doctor in pharmacy, Physiology Department, Faculty of Medicine and Dentistry, University of Valencia, Spain; 5 Chairman of Oral Surgery, Stomatology Department, Faculty of Medicine and Dentistry, University of Valencia, Spain; 6 Chairman of Physiology, Physiology Department, Faculty of Medicine and Dentistry, University of Valencia, Spain

## Abstract

**Background:**

Different methods have been used in order to isolate dental pulp stem cells. The aim of this study was to study the effect of different types of pulp treatment during isolation, under 3% O2 conditions, in the time needed and the efficacy for obtaining dental pulp stem cells.

**Material and Methods:**

One hundred and twenty dental pulps were used to isolate dental pulp stem cells treating the pulp tissue during isolation using 9 different methods, using digestive, disgregation, or mechanical agents, or combining them. The cells were positive for CD133, Oct4, Nestin, Stro-1, CD34 markers, and negative for the hematopoietic cell marker CD-45, thus confirming the presence of mesenchymal stem cells. The efficacy of dental pulp stem cells obtention and the minimum time needed to obtain such cells comparing the 9 different methods was analyzed.

**Results:**

Dental pulp stem cells were obtained from 97 of the 120 pulps used in the study, i.e. 80.8% of the cases. They were obtained with all the methods used except with mechanical fragmentation of the pulp, where no enzymatic digestion was performed. The minimum time needed to isolate dental pulp stem cells was 8 hours, digesting with 2mg/ml EDTA for 10 minutes, 4mg/ml of type I collagenase, 4mg/ml of type II dispase for 40 minutes, 13ng/ml of thermolysine for 40 minutes and sonicating the culture for one minute.

**Conclusions:**

Dental pulp stem cells were obtained in 97 cases from a series of 120 pulps. The time for obtaining dental pulp stem cells was reduced maximally, without compromising the obtention of the cells, by combining digestive, disgregation, and mechanical agents.

**Key words:**Dental pulp stem cells, mesenchymal stem cells, isolation method.

## Introduction

Dental pulp stem cells (DPSC) were first isolated by Gronthos *et al.*(1) in 2000. These cells demonstrated clonologenic capacity and were able to differentiate into odontoblasts and form a dentin-pulp complex when they were implanted subcutaneously in immune-compromised rats. Different factors like culture medium (2,3), O2 pressure (4,5), and type of pulp treatment during isolation (6,7) can influence cell proliferation.

The original method used by Gronthos *et al.* (1) to obtain DPSC was the enzymatic digestion of the dental pulp, previously fragmented, with 3 mg/ml collagenase type I and 4mg/ml of dispase for 90 minutes at 37°C. Many authors (8-14) have used this protocol to obtain DPSC. Other methods, using different digestive agents (15,16) and protocols where the pulp is fragmented without performing any kind of digestion (17) are described. Few studies analyze the influence of pulp treatment during isolation when obtaining DPSC. Karamzadeh *et al.*(6) and Hilkens *et al. * (7) studied the obtention of DPSC by enzymatic digestion of pulp and through mechanical fragmentation. They obtained DPSC with both methodologies; cells were obtained in 3 to 5 days by enzymatic digestion of the pulp and on the fifth day with mechanical fragmentation (6). They observed no differences in terms of proliferation, colony formation or differentiation between the two methods (7). Only Perry *et al.* (18) have used a large series of pulps to study the efficacy of obtention of DPSC. Many publications (2-7) analyze the characteristics and applications of stem cells from dental tissues, but from a clinical point of view, a systematic study of the efficacy of DPSC obtention would be advantageous (18).

Tirino *et al.* (19) in a recent review about strategies and perspectives with DPSC, emphasize the importance of finding methods for reducing the time needed to obtain these cells. From a clinical application viewpoint, an important factor would be the time needed and the efficacy in obtaining DPSC. Our aim was to determine the time needed and the efficacy in obtaining DPSC applying 9 different pulp treatments to 120 pulps while they were being isolated under 3% O2 conditions.

## Material and Methods

A prospective *in vitro* study, approved by the ethics committee of the University of Valencia that fulfilled the Declaration of Helsinki principles, was performed. The patients were informed of the characteristics of the study and freely agreed to cooperate with it, providing the extracted tooth, and signing an informed consent form. One hundred and twenty pulps were extracted from 107 patients, 35 men and 85 women. Their ages ranged between 14 and 67 years.

- Inclusion and exclusion criteria and data recorded

Pulps from teeth without pulpitis or pulp necrosis were included. Pulps from teeth presenting any of the following medical conditions were excluded: pulpitis, apical periodontitis, fractures affecting the dental pulp, and teeth which were fractured during extraction or require odontosection.

The following parameters were recorded from the extracted pulps: gender and age of the patient, date of tooth extraction and cell culture, type of pulp treatment during isolation, efficacy in obtaining DPSC (whether cells were obtained or not), and the time needed to obtain them.

- Extraction, storage, and transport of the dental pulp

The pulp chamber was accessed by cutting the teeth at the cementoenamel junction with a turbine and diamond bur. The dental pulp was extracted with tweezers and stored in Dulbecco’s modified Eagle® culture medium (Invitrogen. Carlsbad, Calif., USA) supplemented with low glucose (1mg/ml) and antibiotics (penicillin and streptomycin).

- DPSC isolation

Dental pulp was fragmentized with a nº10 blade scalpel. Fragments were collected and treated with 9 different methods (methods A-I), some are described in the literature and others were original, with the aim of reducing the time needed to obtain DPSC.

The methods were the following: digestion with collagenase, dispase Sigma-Aldrich (St. Louis, Mo.,USA) or termolysine Vitacyte (Indianapolis, In., USA). Disgregation (with EDTA), mechanical treatment of the pulp (fragmentation with scalpel or sonication) or a combination of the methods mentioned above.  Table 1 details the 9 treatments and the number of pulps used in each method.

**Table 1:**
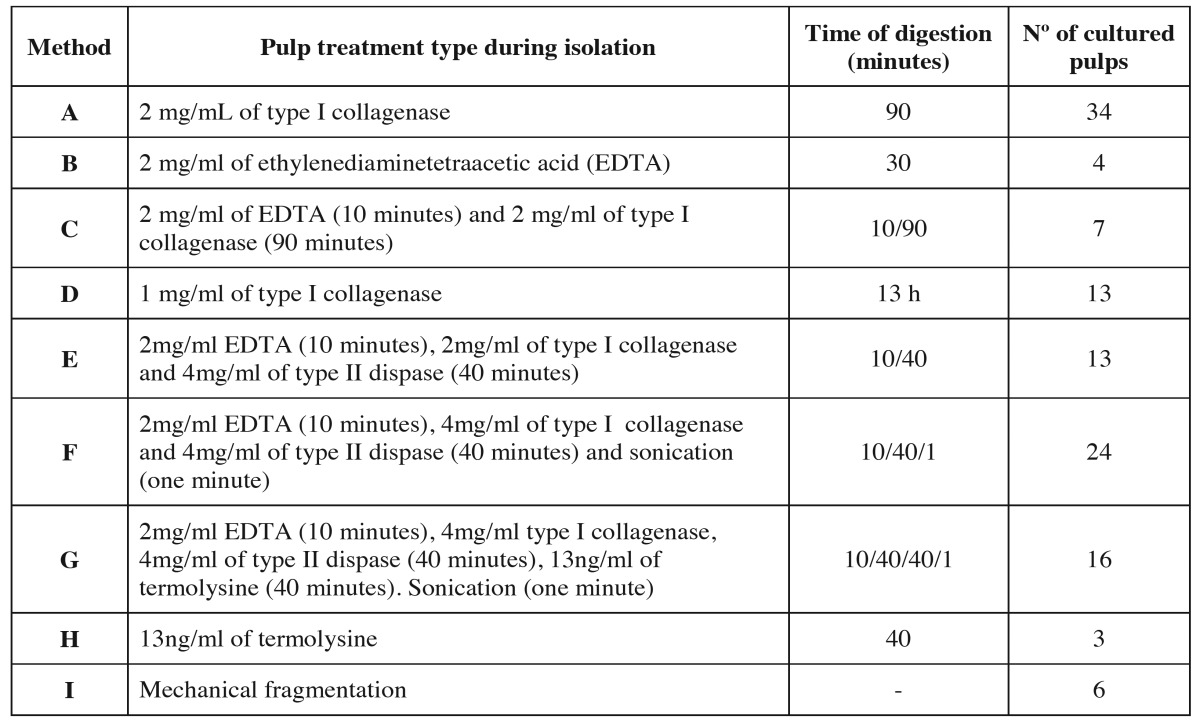
Type of pulp treatment during isolation, time of pulp tissue digestion/disgregation and number of pulps used in each method.

Conditions during isolation were 37°C, 5% CO2 and 3% O2. After pulp digestion, it was centrifuged at 1000 g for 2 minutes, the pellet was collected and cultured with complete culture medium. (DMEM supplement with low glucose (1mg/ml) 90% (v/v) and bovine fetal serum 10% (v/v) at 37°C, 5% CO2 and 3% O2.

**Figure 1:**
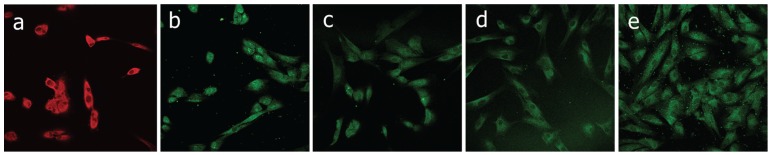
Characterization of the stem cells from the dental pulp by confocal microscopy. a) Image 40x of the marker CD-133. b) Image 40x of the marker Oct4. c) Image 40x of the marker Nestin. d) Image 40x of the marker Stro-1. e) Image 40x of the marker CD-34. The DPSC were negative for the marker CD-45.

The presence of DPSC was confirmed as these cells were positive for the markers CD133, Oct4, Nestin, Stro-1, CD34 and negative for the hematopoietic marker CD45. Characterization was performed with cells obtained in method A. Figure 1a-e.

- Time required to obtain DPSC

The time point at which digestion and/or disgregation of the pulps was complete was termed “0” at which point the time needed to obtain DPSC was recorded.

Cells were considered to be present when a fibroblastic morphology cell, as Friedenstein *et al.* (20) described mesenchymal stem cells, appeared from a pulp fragment.

## Results

**Figure 2:**
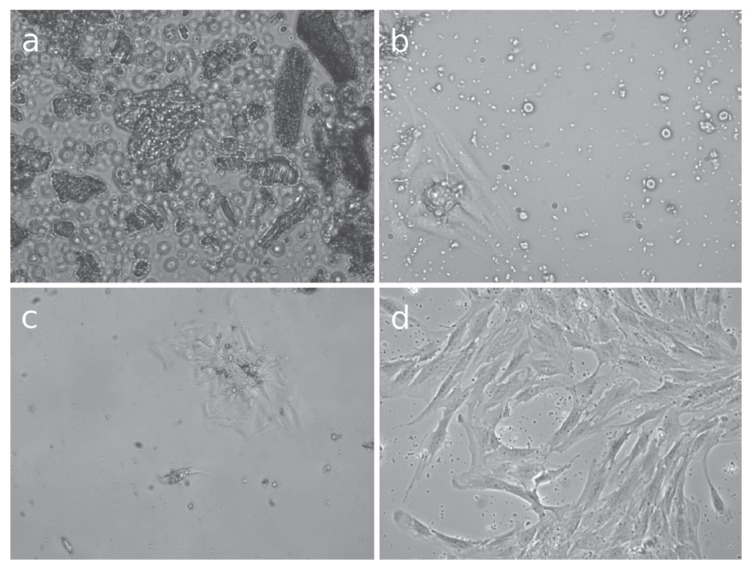
Optical microscopy images of the obtainment of Dental pulp stem cells, digestion with 1 mg/mL of collagenase for 13 hours. a) Image of the pulp after digestion magnified 400x. Pulp fragments in suspension can be observed. b) Image of Dental pulp stem cells after 24 hours of culture magnified 400x. Cells are released from a pulp fragment and adhere to the culture plate. c) Image of Dental pulp stem cells after 48 hours of culture magnified 200x. The number of cells is higher. d) Image after 17 days magnified 400x The culture plate is subconfluent.

 Table 2 details the efficacy and the time needed to obtain DPSC with each method. One hundred and twenty pulps were used for the study, and DPSC were obtained in 97 cases, so the efficacy in obtaining DPSC was 80.8%. DPSC were obtained with all the methods used except fragmentation of the pulp, where no pulp digestion was carried out. The minimum time required to obtain DPSC was 8 hours with method G (2mg/ml EDTA for 10 minutes, 4mg/ml of collagenase type I, 4mg/ml of dispase type II for 40 minutes and 13ng/ml of termolysine for 40 minutes, sonication of the culture for one minute). However, with this method, cells were obtained with all the pulps (16 pulps), so cell viability does not seem to be reduced by the combination of different digestive, disgregant, and mechanical agents. Method A required the maximum time needed to obtain cells, which was 14 days. The minimum efficacy, except for mechanical fragmentation, was observed with method H (digestion with 13ng/ml of thermolysine), where an efficacy of 33% was observed, but only 3 pulps were used with this method. The digestion agent acted the longest with method G (digestion with collagenase for 13 hours). With this method, DPSC where obtained in 84% of the cultured pulps (11 pulps out of 13). As seen in this method, a long digestion period affords cell viability. Figure 2 shows optical microscopy images of different time period in which DPSC were obtained with this method.

**Table 2:**
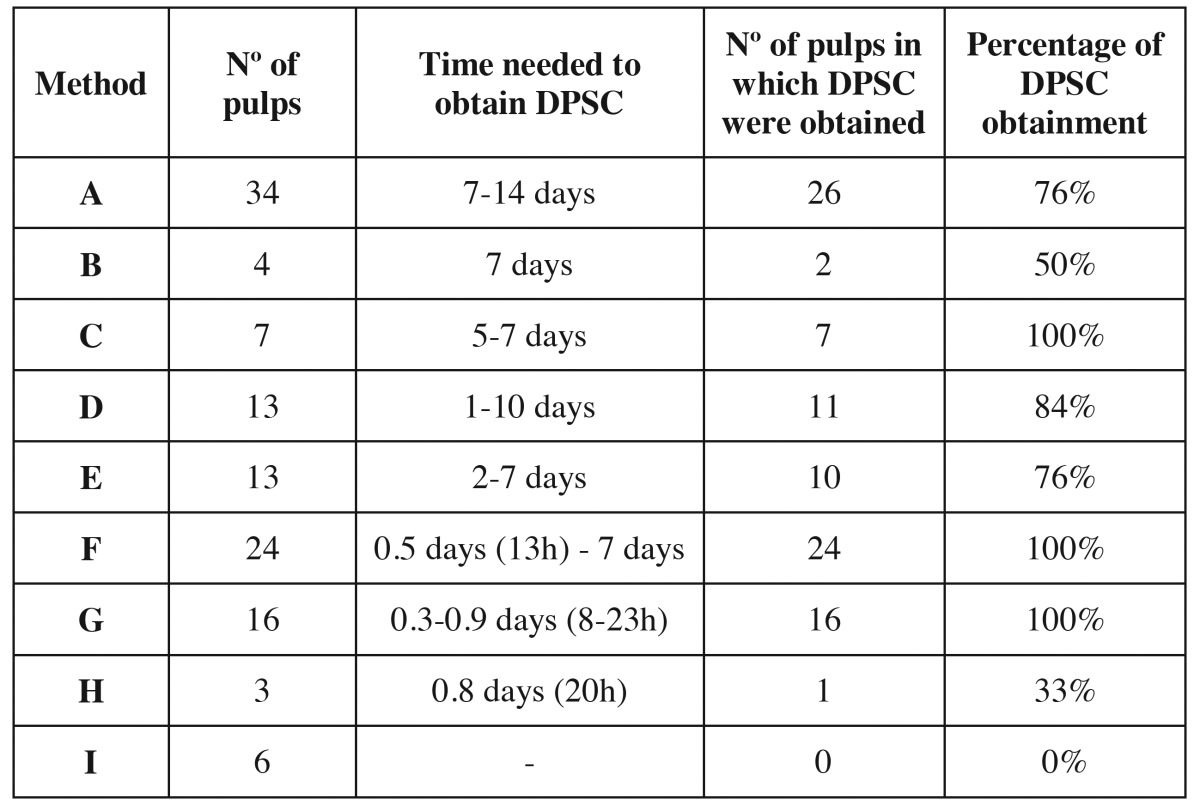
Number of pulps used in each method, number of pulps from which cells were obtained, time and efficacy of Dental pulp stem cells obtainment.

## Discussion

The present study aimed to verify the efficacy and the time needed to obtain DPSC, and to obtain cells in the minimum time require comparing 9 different methodologies. Dental pulp is an easily harvested tissue to obtain stem cells; the percentage and time needed to obtain the cells, are both important to apply DPSC clinically. In the present study we ascertained that by combining different disgregating (EDTA), digestive (collagenase, dispase and termolysine) and mechanical (sonication) agents, the time needed to obtain DPSC diminishes. The minimum time require to obtain DPSC was achieved by using 2mg/ml EDTA for 10 minutes, 4mg/ml of type I collagenase, 4mg/ml type II dispase for 40 minutes, 13ng/ml of thermolysine for 40 minutes and sonicating the culture for one minute. DPSC appeared after 8 hours using this method. Nonetheless, when different agents were combined, the obtention percentage was not compromised. There are only 2 studies comparing 2 pulp treatments during isolation. They compared the enzymatic digestion of pulp tissue using collagenase and dispase and mechanical fragmentation. Karamzadeh *et al.* (6) obtained DPSC with both methodologies and observed them 3 to 5 days after culture by digestion, and on the fifth day by fragmentation of the pulp. The cells were morphologically the same using both methods. Hilkens *et al.* (7) did not observe differences in proliferation, colony forming or differentiation when using the 2 methods.

There is only one study, performed by Perry *et al.* (18), that analyzes an extended series of dental pulps to assess the efficacy of obtention of DPSC. They cultured 40 pulps and observed DPSC 24 hours after culture. Cell growth was seen in 31 cases (77.5% of efficacy), fungal contamination in 7 and no cell growing in 2 cases. In the present study, a similar percentage of cell obtention (80.8%) was observed. There was no fungal contamination, and unlike the study by Perry *et al.* (18), our culture medium did not contain antifungal agents. These authors (18) believe the fungal contamination was due to endogenous host causes rather than postexodoncy contamination. According to these data, the presence or absence of antifungal agents in the culture medium does not influence contamination (18).

Most studies do not detail the time needed to obtain DPSC or the method used to obtain them. Only a few publications, performed by Laino *et al.* (21) and Takeda *et al.* (8), detail that DPSC were obtained 5 days after using the original protocol described by Gronthos *et al.* (1) (3 mg/ml collagenase type I and 4 mg/ml of dispase for 90 minutes). In the present study, the minimum time needed to obtain DPSC was 8 hours. No other publication reports a shorter time.

There are different methods for isolating DPSC: Waddinton *et al.* (15) used 4 mg/ml of collagenase/dispase for one hour. Another method is to digest with type I and II collagenase and with thermolisyne for 40 minutes at 37 Cº (18). Zang *et al.* (16) used 3 mg/ml of type I collagenase. Sasaki *et al.* (22) cultured rat DPSC digesting with 0.25 % of tripsin/EDTA for 15 minutes. Other authors (23-25) also used the original protocol established by Gronthos *et al.* (1) but they reduced the digestion time to one hour. One of the protocols performed in the present study was to digest the pulp with 2 mg/ml type I collagenase for 90 minutes at 37ºC; this method was previously used in another study (26). Human DPSC were also obtained by treating the pulp with agents not described in the literature, like EDTA, only digesting with thermolysine or sonicating the culture after digestion. However, DPSC could not be obtained by mechanical fragmentation of the pulp. This result differs from another study (17). It must be highlighted that, in the present study, DPSC could be obtained by disgregation of the pulp with EDTA, which is a chelation agent of calcium that breaks down the desmosomas that blind the cells but is not a digestive agent which digest the intercellular protein network. It is also important that cell viability was observed in less time when using 1mg/ml of collagenase for a long period of time (13 hours). This method is based on corneal cell cultures (27). The time needed to obtain DPSC in our study was influenced by the physiological conditions of O2 (3%) during isolation and culture. It has been demonstrated that there is a high proliferation rate of pulp cells at 3% (4) or 5% (5) O2 than at 20% (environmental conditions).

## Conclusions

DPSC were obtained from 97 of 120 pulps in a case series (80.8% of efficacy). After comparing 9 different treatments during isolation, the minimum time needed to obtain DPSC was 8 hours after digestion of the pulp tissue with 2mg/ml EDTA for 10 minutes, 4mg/ml of collagenase type I, 4mg/ml of dispase type II for 40 minutes and 13ng/ml of termolysine for 40 minutes, and sonicating the culture for one minute.
